# Identification of molecular biomarkers for pancreatic cancer with mRMR shortest path method

**DOI:** 10.18632/oncotarget.18186

**Published:** 2017-05-25

**Authors:** Shuhua Shen, Tuantuan Gui, Chengcheng Ma

**Affiliations:** ^1^ Zhejiang Provincial Hospital of Traditional Chinese Medicine, Hangzhou, China; ^2^ Shanghai Smartquerier Biotechnology Co., Ltd, Shanghai, China; ^3^ CAS-MPG Partner Institute for Computational Biology, Shanghai Institutes for Biological Sciences, Chinese Academy of Sciences, Shanghai, China; ^4^ Shanghai Center for Bioinformatics Technology, Shanghai, China

**Keywords:** minimum-redundancy-maximum-relevance (mRMR), pancreatic cancer, biomarker

## Abstract

The high mortality rate of pancreatic cancer makes it one of the most studied diseases among all cancer types. Many researches have been conducted to understand the mechanism underlying its emergence and pathogenesis of this disease. Here, by using minimum-redundancy-maximum-relevance (mRMR) method, we studied a set of transcriptome data of pancreatic cancer. As we gradually added features to achieve the most accurate classification results of Jackknife, a gene set of 9 genes was identified. They were *NHS*, *SCML2*, *LAMC2*, *S100P*, *COL17A1*, *AMIGO2*, *PTPRR*, *KPNA7* and *KCNN4*. Through STRING 2.0 protein-protein interactions (PPIs) analysis, 40 proteins were identified in the shortest paths between genes in the gene set, 30 of them passed the permutation test, which indicated they were hubs in the background network. Those genes in the protein-protein interaction network were enriched to 37 functional modules, such as: negative regulation of transcription from RNA polymerase II promoter, negative regulation of *ERK1* and *ERK2* cascade and *BMP* signaling pathway. Our study indicated new mechanism of pancreatic cancer, suggesting potential therapeutic targets for further study.

## INTRODUCTION

Pancreatic cancer is one of the most lethal diseases among all cancer types, leading to about 79,400 deaths in China [1] and 330,400 deaths worldwide [2]. The five-year survival rate is only 2–7% [3, 4]. This poor outcome could be largely due to the late diagnosis. The mechanism underlying its progression is still unclear. The expression profiles of pancreatic cancer had been widely studied, revealing several molecular factors affecting various aspects of pancreatic cancer [5]. Terris et al. found four genes — caveolin 1, glypican 1, growth arrest-specific 6 protein, cysteine-rich angiogenic inducer 61 were associated with the pathogenesis of pancreatic cancer and possible early stage pancreatic cancer indicators [6]. For some patients, *PCK1, SFRP2* were identified as potential metastasis markers in a study comparing the expression profiles primary and metastasis pancreatic cancer [7]. *GSTT1, TOP2A, CASP3* and *ABCC2* had been found to possess gemcitabine sensitivity predictive properties [8]. These studies usually adopted differentially expressed genes (DEG) methods. This means the studies considered the relevance between expression levels and certain phenotype separately, ignoring the relationships between the genes. These methods would bring redundancies to the findings, mixing the most representative genes into the bulk results.

Feature selection often means the process of maximizing the classification accuracy with the combination of the selected features integrating into a classification model. To that end, people select the features passing certain relevance threshold. Relevance is usually characterized in terms of correlation or mutual information. But many genes work closely as a functional module. The interactions among them may contribute to class distinctions. However, combinations of individually good features are not necessarily a good gene set representing the whole picture underlying the biological processes [9]. Minimum-redundancy-maximum-relevance (mRMR) had been widely used in several biological fields such as predicting lysine ubiquitination [10], protein-protein interactions [11] and HIV Progression-Related Genes [12]. This method considers the associations between the features and the target phenotype, together with the inner relationships among the features. Comparing with the other methods, mRMR showed better classification accuracy [13].

The proteins work together to form functional modules. Investigating the disease candidate genes should consider these interactions for better understanding how the candidates function. Among the interaction databases, STRING (Search Tool for the Retrieval of Interacting Genes) [14] is most frequently used because of its millions interactions and the high quality scoring system. With this powerful data source, we can restore the overall functional impact of the genes of our interest.

In this study, we performed a Minimum-redundancy-maximum-relevance (mRMR) based transcriptome study. The objective was to find a set of genes which best classifying these two types of samples, explaining some mechanisms of the pathogenesis of pancreatic cancer. Based on graphic analysis [15] on STRING PPIs network we further identified pancreatic cancer association genes and functional modules worthy for further experimental studies.

## RESULTS

### Gene probes identified by mRMR-IFS

**Figure 1:**
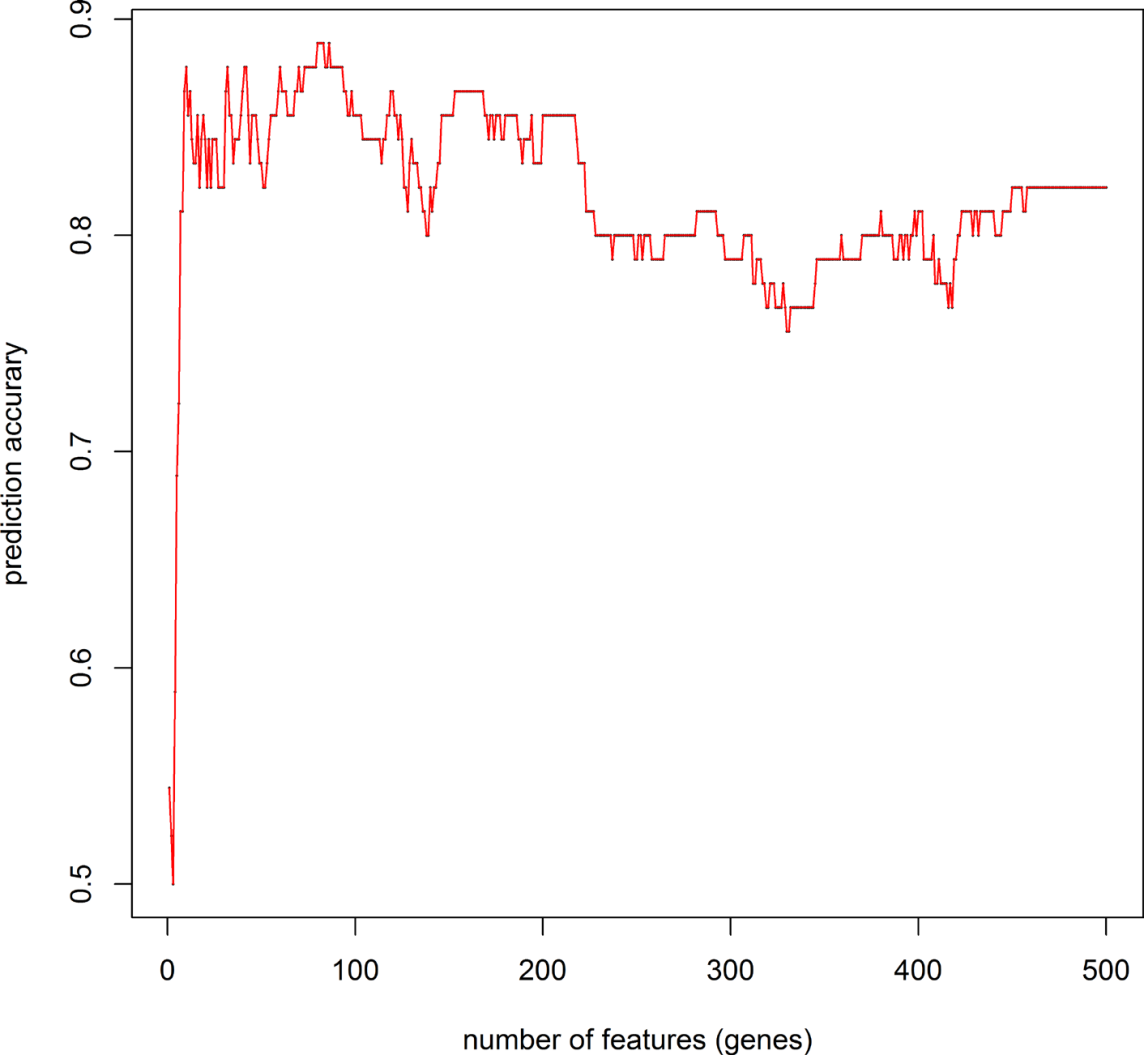
IFS curve to determine the number of features used in prediction We used an IFS curve to determine the number of features finally used in the mRMR feature selection. Prediction accuracy reached its second maximum value at 10 gene probes. The x-axis indicates the number of probes used for classification, and the y-axis is the prediction accuracy.

**Figure 2:**
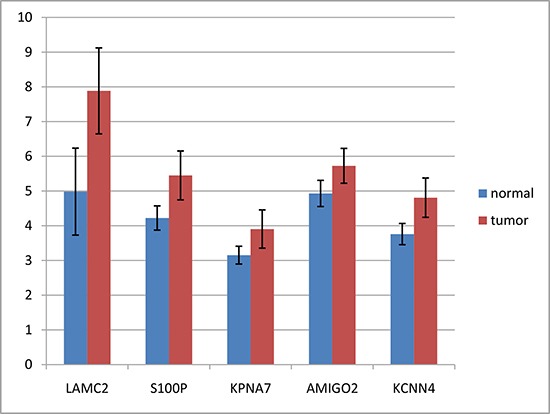
Expression differences of LAMC2, S100P, KPNA7, AMIGO2 and KCNN4 between tumors and non-tumors Format: PNG This figure shows the expression differences of LAMC2, S100P, KPNA7 and AMIGO2 between tumors and non-tumors, separately. Error bars indicate standard errors.

We retrieved 45 pancreatic cancer and 45 non-tumor samples’ gene expression profiles from GEO (GSE28735) consisting 28,869 probes. We used mRMR-IFS method to do feature selection and used K-nearest-neighbor model to do phenotype classification (see Methods). We adopted K-nearest-neighbor model and jackknife validation, and calculated the classification accuracy of 1 to 500 probes (Figure 1). We found a set of 10 probes with the accuracy of 0.88, which is close to the highest accuracy of 0.89 with 80 probes. The 10 gene probes set would be more representative than 80 gene probes set, so we choose 10 gene probes (Table 1). The differential expression of *LAMC2, S100P, KPNA7, AMIGO2* and *KCNN4* had also been reported in other studies [16–20] (Figure 2). Some genes had been reported to be related to PDAC, such as *LAMC2*, *S100P* and *KPNA7* [18, 21, 22]. We also identified novel pancreatic cancer genes, such as *SCML2*, *COL17A1*, *AMIGO2*, *PTPRR*, suggesting our method might be able to predict novel PDAC-related genes.

**Table 1:** Top 10 of the genes by betweenness in the shortest paths

Probe ID	seqname	STRAND	START	STOP	Gene Symbol	mRMR score
8166266	chrX	+	17393543	17754114	NHS	0.26468
8171561	chrX	−	18257433	18372847	SCML2	0.262552
7908072	chr1	+	1.83E+08	1.83E+08	LAMC2	0.280165
8093950	chr4	+	6694796	6698897	S100P	0.300541
8017098	chr17	−	56736510	56736657		0.270637
7936144	chr10	−	1.06E+08	1.06E+08	COL17A1	0.28748
7962579	chr12	−	47469490	47473734	AMIGO2	0.270462
7964907	chr12	−	71031853	71314586	PTPRR	0.232587
8141263	chr7	−	98775543	98805089	KPNA7	0.188924
8037408	chr19	−	44270685	44285409	KCNN4	0.817682

### A PPI sub-network of the genes

**Figure 3:**
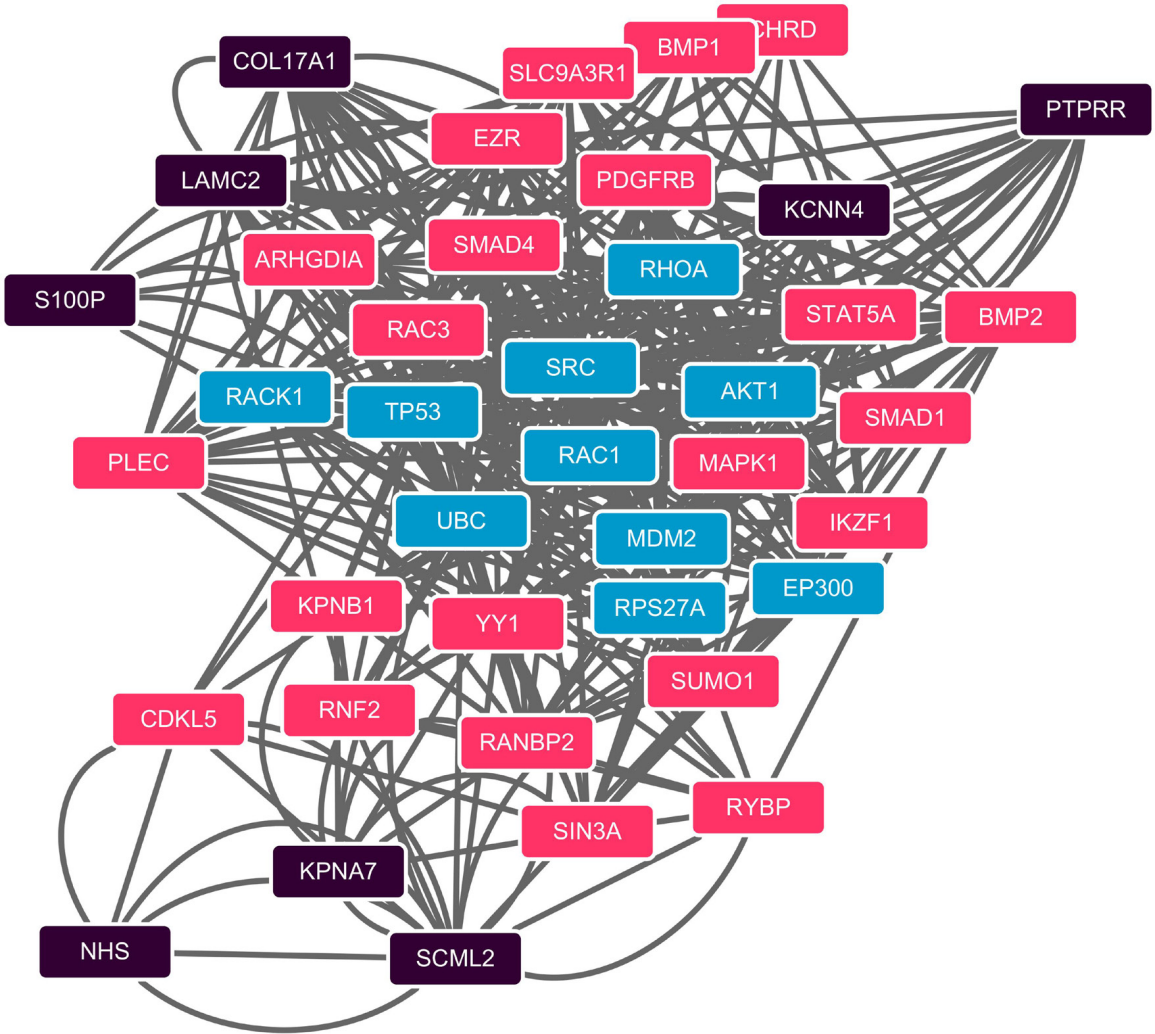
PPI network of shortest paths among 40 computational method identified proteins Shortest paths between each pair of the 8 proteins (black) which from the 40 computational method selected proteins were identified in the STRING PPI network. Proteins in black are the 8 identified genes using the computational method which also present in the STRING PPI network; red ones are shortest paths proteins passed the permutation test; blue are not passed ones.

We further built up an undirected network using PPIs from STRING [14]. The protein pairs with PPI score greater than 0.8 were used to form high confidence network. From the 10 gene probes identified by mRMR-IFS, we found 9 genes corresponding to 27 proteins in STRING. 8 proteins were in the high confidence network. We computed the shortest path of every pairs of proteins using the Dijkstra's algorithm [15]. The shortest paths were integrated into a sub-network (Figure 3), and the sub-network contains 51 protein-protein interactions involving 40 proteins. We conducted a permutation test to evaluate the significance of betweenness of the proteins against background network. 30 proteins passing the test were selected and ranked according to their betweenesses (Supplementary Table 1). Among the betweenesses, MAPK1's had the largest, which was 14, indicating there were at least 7 shortest paths going through this gene.

### Functional enrichment analysis of the genes

Using DAVID, we implemented GO functional enrichment analysis and KEGG pathway analysis with the 10 probes. Results showed that these genes were significantly enriched in the cell adhesion in organelle (Supplementary Table 2). Only one KEGG pathway was significantly enriched (hsa04974: Protein digestion and absorption) (*p*-value = 0.038, Supplementary Table 3).

We also performed KEGG pathway and GO functional enrichment with the 30 hub genes on the shortest paths. The GO results showed that many genes were significantly enriched in the modules of negative regulation of transcription from RNA polymerase II promoter (Supplementary Table 4). And the KEGG pathway results showed that these genes were significantly enriched in the TGF-beta signaling pathway (hsa05212: Pancreatic cancer, *p*-value=3.08E-05, Supplementary Table 5).

## DISCUSSION

In a previous study, Zhang et al. identified 277 genes to be differentially expressed with this set of data [23]. By our approach, a more compact set of features was identified (Supplementary Table 4) with high classification accuracy.

Among the more than 20,000 probes in the transcriptome data, we selected 10 probes corresponding to 9 genes as the most optimized predictors. They are *NHS, SCML2, LAMC2, S100P, COL17A1, AMIGO2, PTPRR, KPNA7* and *KCNN4*. Some of them had been proved to be associated with pancreatic cancer.

*LAMC2* (Laminin subunit gamma-2) Laminins are extracellular matrix glycoproteins. Studies showed that they are involved in many biological processes including cell adhesion, differentiation, and metastasis [24–26]. The overexpression of LAMC2 had been shown to be a predictive marker of pancreatic cancer [21]. Another microarray study also found it overexpressed in PDAC tumor epithelia. Moreover, its expression level negatively correlated with survival [27]. Nerve invasion is a prominent feature of pancreatic cancer. In a study with cell line, mouse model and patients’ surgical tissues, overexpression of LAMC2 was observed to be positively associated with nerve invasion distance [28].

*S100P* (S100 calcium binding protein P) is a member of S100 family of proteins. S100 regulates cell cycle progression and differentiation [29]. Microarray study had shown it specifically expressed in the neoplastic epithelium of pancreatic cancer [22]. The expression level of S100P is correlated with the rates of cell proliferation, survival, migration and invasion, which makes S100P protein a major promoting factor in the pathogenesis of pancreatic cancer [30]. The abnormal expression might be because of hypomethylation [31]. Overexpression of S100P is an early marker of pancreatic cancer, which down-regulates the levels of cytoskeletal proteins, which disrupts the actin cytoskeleton network and changes in the phosphorylation status of cofilin. S100P also un-regulates expression of two cellular invasion factors S100A6 and aspartic protease cathepsin [32].

*AMIGO2* (Adhesion Molecule With Ig Like Domain 2) also named as DEGA (Differentially expressed in gastric adenocarcinomas). As its name DEGA, it may induce several deterious alterations including aneuploidy and abnormal adhesion in gastric cancers [33, 34]. Antibodies against AMIGO2 had been proved to be effective to pancreatic cancer in xenograft models [17].

*KPNA7* (karyopherin subunit alpha 7) is a member of importin α family. *In vitro* experiments had demonstrated that *KPNA7* was up-regulated in pancreatic cancer. Silencing KPNA7 could increase the level of p21, promote G1 arrest, and increase autophagy [18]. It is an important factor promoting the malignant of pancreatic cancer.

*KCNN4* (potassium calcium-activated channel subfamily N member 4) consists Ca^2+^ activated voltage-independent K^+^ channel [35]. Ca^2+^-activated K^+^ channels are involved in anion and K^+^ transport in stimulated pancreatic cells [36]. *In vitro* study had shown that blocking the channels could inhibit the growth of pancreatic cancer, which suggested the important role of them in the proliferation of pancreatic cancer [16].

## MATERIALS AND METHODS

### Dataset

The microarray gene expression profiling dataset was downloaded from NCBI Gene Expression Omnibus (accession no.: GSE28735). The dataset contains 45 tumor and 45 non-tumor patients with pancreatic ductal adenocarcinoma (PDAC) [23].

### Feature selection

To rank the importance of the features that best distinguish pancreatic ductal adenocarcinoma tumor from normal adjacent tissues, we applied mRMR method, which ranks the features according to their relevance to the target phenotypes minus the redundancy between the features [37]. In our study, we used R package mRMRe to implement mRMR [38]. In mRMRe, both relevance and redundancy are quantified by mutual information (MI):

$$\iint \text{p}(\text{x},\text{y})\text{log}\frac{\text{p}(\text{x},\text{y})}{\text{p}(\text{x})\text{p}(\text{y})}\text{dxdy}$$  1

Where x and y are two variables to be tested, p(x) and p(y) are the marginal probabilistic densities, and p(x, y) is their joint probabilistic density, and I(x, y) represents the MI.

Let X = {x_1_,.....,x_n_} denote the set of gene probes (input features), and let y denote the phenotype (input target). Given the feature with highest MI between the phenotype x_i_, the set of ranked features S is initialized with x_i_. Next, the best balance between maximal relevance and minimum redundancy in the remaining feature x_j_ is added to S. It is selected by maximizing the score q according to the following equation:

$$\text{q }{=\text{I}(\text{x}}_{\text{j},}\text{y})-\frac{1}{|\text{S|}}\sum_{\text{Xk}\in \text{S}}{\text{I}(\text{x}}_{\text{j}}{,\text{x}}_{\text{k}})$$ 2

The selection step is repeated until a desirable number of ranked features N, which was 500 in our study.

To determine an appropriate subset of the ranked feature list, we chose incremental feature selection (IFS) to determine the most suitable number of the genes in the feature subset s_i_ [39]:

$${\text{S}}_{i}=\left\{{\text{f}}_{1},{\text{f}}_{2},\ldots ,{\text{f}}_{i}\right\}(1\leq \text{i}\leq \text{N})$$ 3

For example, N is 500, then the first feature subset is s_1_ = {f_1_}, the second feature subset is s_2_ = {f_1_, f_2_}, and the last feature subset is S_N_ = {f_1_, f_1_,...f_500_}. The feature subset with the best prediction accuracy is selected.

### Prediction engine

We used k-nearest neighbor method to predict the phenotype of each individual. In our study, the distance between two individuals was defined according to Chou and coworkers [40, 41]:

$${\text{D}(\text{i}}_{2}{,\text{i}}_{2})=1-\frac{{\text{e}}_{1}-{\text{e}}_{2}}{\left|{\text{e}}_{1}|\cdot |{\text{e}}_{2}\right|}$$ 4

Where i_1_ and i_2_ represent two individuals, D refers to the distance between the two individuals, and e_1_ and e_1_ are vectors of selected feature sets (expression levels of selected genes) of the two individuals.

### Validation method

Independent dataset test, subsampling test, and jackknife test are three validation methods that are often used in statistical model validation. Comparing to other two validation methods jackknife test is better at avoiding the arbitrariness that exists in the independent dataset and subsampling test [40, 42, 43]. In jackknife test, both the training dataset and testing dataset are open. Each sample will be in turn moved between the training dataset and testing dataset.

The prediction accuracy was formulated as:

$$\text{Accuracy }=\frac{\text{TP}+\text{TN}}{\text{TP}+\text{TN}+\text{Fp}+\text{FN}}$$ 5

Where TP represents the number of true positives, TN represents the number of true negatives, FP represents the number of false positives, and FN represents the number of false negatives.

### Graphics approach and shortest paths tracing

The initial weighted PPIs network was retrieved from STRING(version 10) [14], and used to constructed a graph G(V,E). The database contains known and predicted protein interactions, which provides intuitive insights and overall structure properties to study complex biological systems. Based on the PPIs network, we used Dijkstra's algorithm [15] to identify the shortest path between any pair of proteins that were identified by mRMR-IFS. The visualization of subnetwork with the shortest paths was done by Cytoscape [44].

### Permutation test

To test whether the 40 shortest path genes were hubs in the background network, we conducted a permutation test. Occurrences of the 40 proteins were counted up in the shortest paths between randomly selected 8 proteins when they had higher betweenness than that of shortest path genes. This process was repeated 1000 times. The *p*-value was calculated as the proportion of the occurrence times of the 40 proteins in 1000 permutations. Shortest path genes with a *p*-value below 0.05 were considered as significant pancreatic cancer related in this study.

### Pathway enrichment analysis

We used the functional annotation tool DAVID [45] for KEGG pathway enrichment and GO functional enrichment analysis. Significant functional modules were selected with a corrected *p*-value < 0.05.

## CONCLUSIONS

In this study, we implemented a minimum-redundancy-maximum-relevance (mRMR) based transcriptional profile study to present a comprehensive view of the features in pancreatic cancer. We identified *NHS, SCML2, LAMC2, S100P, COL17A1, AMIGO2, PTPRR, KPNA7* and *KCNN4* as closely related genes to the disease. Some of them had been validated *in vitro* and/or *in vivo*. From the functional analysis of PPIs network, RNA polymerase II and growth factor function showed importance to this disease. In conclusion, our method provided solid and novel insights to this mortal disease, suggesting several genes and functions that worth further investigations.

## SUPPLEMENTARY MATERIALS FIGURES AND TABLES












