# Supplementary material for: Identification of molecular biomarkers for pancreatic cancer with mRMR shortest path method
Source: Oncotarget. 2017 May 25;8(25):41432–9. doi: 10.18632/oncotarget.18186 (PMC5522256; doi:10.18632/oncotarget.18186)
Supplement: Supplementary file 1 [file oncotarget-08-41432-s001.pdf]

Identification of molecular biomarkers for pancreatic cancer with mRMR shortest path method

Supplementary Materials

Supplementary Table 1: Proteins ranked by the betweenesses, which also passed the permutation test as described in methods. See Supplementary_Table_1

Supplementary Table 2: The GO enrichment of the 10 machine learning method identified features. See Supplementary_Table_2

Supplementary Table 3: The KEGG pathway enrichment of 10 machine learning method identified features. See Supplementary_Table_3

Supplementary Table 4: The GO enrichment of the 30 shortest path identified features, which passed permutation test. See Supplementary_Table_4

Supplementary Table 5: The KEGG pathway enrichment of the 30 shortest path identified features, which passed permutation test. See Supplementary_Table_5
